# Knotted ureteral single‐J stent in a patient with ureterocutaneostomy

**DOI:** 10.1002/iju5.12355

**Published:** 2021-08-05

**Authors:** Yuhei Koike, Fumihiko Urabe, Kosuke Iwatani, Yuto Nukariya, Masatoshi Tanaka, Kojiro Tashiro, Takahiro Kimura, Shunsuke Tsuzuki, Shin Egawa

**Affiliations:** ^1^ Department of Urology The Jikei University School of Medicine Tokyo Japan

**Keywords:** knotted ureteral single‐J stent

## Abstract

**Introduction:**

Although ureteral stent catheterization is a common procedure in urological practice, knotting of a ureteral catheter is a very rare complication.

**Case presentation:**

A 62‐year‐old man underwent cystectomy and ureterocutaneostomy for bladder cancer. The ureteral single‐J stent was changed every month postoperatively without complications. However, at postoperative 30 months, resistance was experienced while adjusting the stent position after the exchange. Abdominal radiography revealed knotting of the stent at the right renal pelvis. The knotted stent was percutaneously removed because the patient had only one functioning kidney. A nephrostomy tract was established and the ureteral stent was exchanged. No complications occurred in the perioperative period.

**Conclusion:**

We encountered a case of a knotted ureteral single‐J stent in a man treated with ureterocutaneostomy. In this case, resistance was noticed during extraction; the possibility of stent knotting should always be considered, and an appropriate removal strategy must be planned.

**Keynote message**

Knotting of the ureteral single‐J stent is a very rare complication. However, in case of resistance during extraction, the possibility of stent knotting must be considered.

## Introduction

Zimskind *et␣al*. first reported the use of ureteral stents for over five decades.^1^ Since then, advances in surgical techniques and stent materials have led to an increase in the indications for its use. Although ureteral stent use can cause various complications,^2^ stent knotting in the renal pelvic or ureter is a rare complication, which makes the extraction of the knotted stent very challenging. Herein, we present a case of a single‐J ureteral stent knotting in a patient treated with ureterocutaneostomy.

## Case presentation

A 62‐year‐old man underwent radical cystectomy for invasive bladder cancer. As retroperitoneal invasion was suspected during surgery, urinary diversion was converted to ureterocutaneostomy. The pathological diagnosis of the resected bladder tumor was high‐grade urothelial carcinoma with the plasmacytoid variant, pT4aN0M0. Based on pathological examination results, three cycles of adjuvant chemotherapy (gemcitabine/cisplatin) were administered. Twenty months after the adjuvant chemotherapy, peritoneal dissemination was observed. Therefore, salvage chemotherapy (gemcitabine/carboplatin) was instituted, and it was ongoing.

**Fig. 1:**
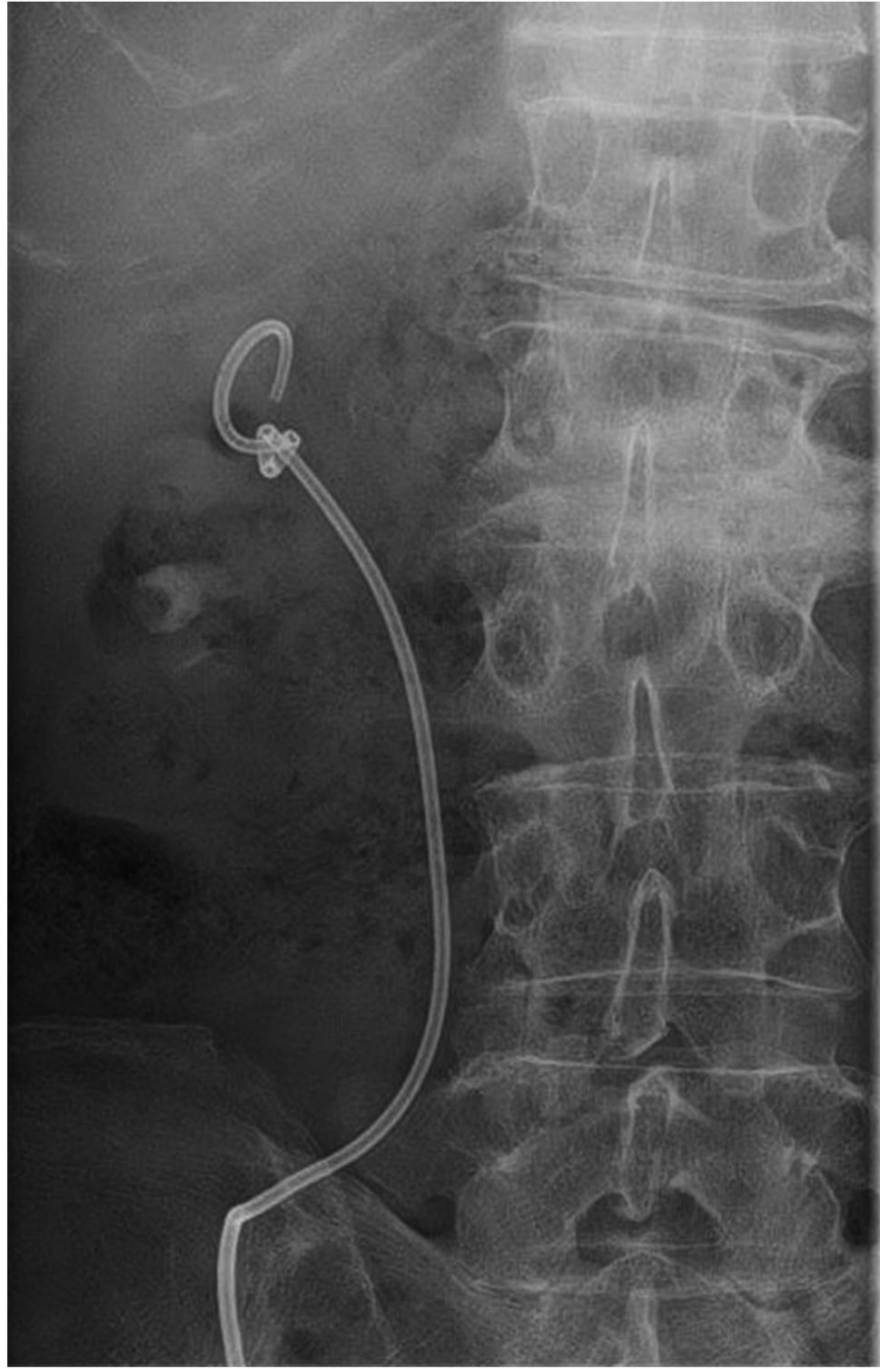
Fluoroscopic image of the knotted stent in the right pelvis.

**Fig. 2:**
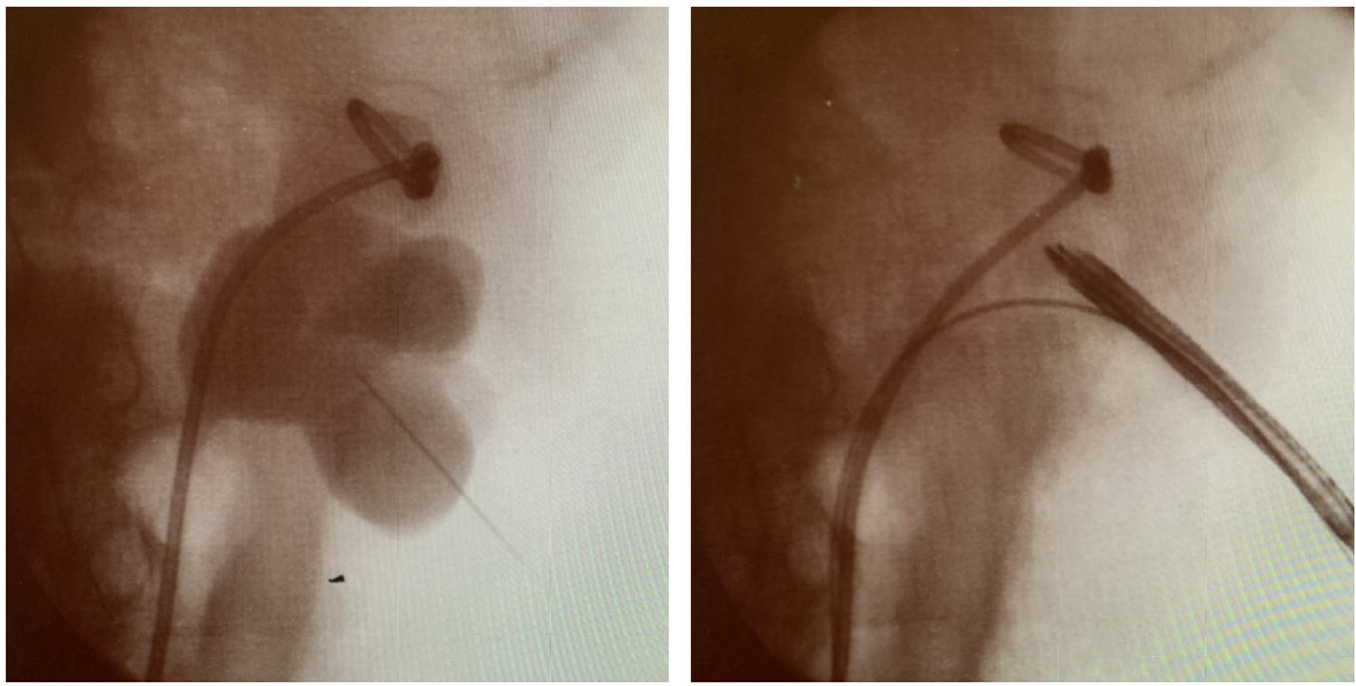
The stent was removed percutaneously through lower renal calix.

**Fig. 3:**
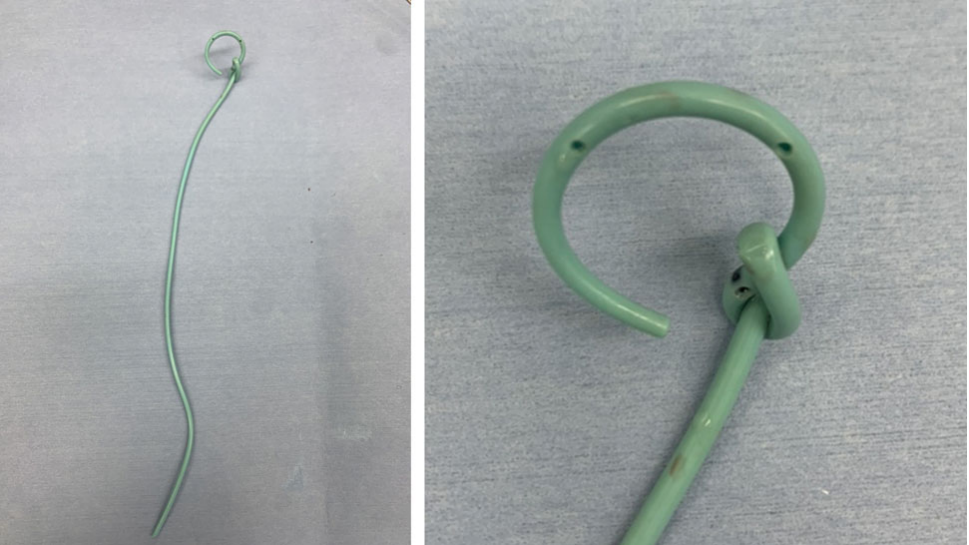
The knotted end of the stent following removal from the patient.

In the outpatient clinic, a 7Fr single‐J stent inserted into the right side of the kidney was changed every month by the physician in charge without any imaging guidance, and no complications had been encountered. However, at postoperative 31 months, the patient visited the outpatient clinic as the length of the single‐J stent from the cutaneoustomy was longer than usual. The single‐j stent was exchanged without an imaging guidance, as usual, by a different clinician. Given the restriction to stent movement during the adjustment of the depth of the single‐J stent, the precise reason was diagnosed using radiography, and the stent was found to be knotted in the right renal pelvis (Fig. 1). First, we attempted to gently retrieve the knotted stent; however, this was not possible because the width of the knotted stent was greater than that of the upper ureter. Next, under imaging guidance, we used a (0.038 inches) guide‐wire to erect the single‐J stent; however, the guide‐wire did not pass through the knotted single‐J stent. As the patient had only one functional kidney, to avoid additional complications, a retrograde approach was ruled out. The distal of knotting stent was migrated into the ureter, which caused the Society of Fetal Urology grade 2 hydronephrosis. Thus, first, ultrasound‐guided percutaneous puncture was performed and the track was dilated with metal dilators. The renal pelvic was carefully observed with a nephroscope, then the tract was additionally dilated with the metal dilators. After a safety guide‐wire was placed, flexible cystoscope was inserted through (along with) another guide‐wire. Finally, the end of the stent was grabbed with a foreign body forceps and removed (Fig. 2). A new single‐J stent was inserted, after which no complications have occurred. As shown in Figure 3, the knotted end of the stent was tight.

## Discussion

The increasing use of ureteral stents in urological practice has increased the frequency of complications. However, knotting of an indwelling ureteral stent is still a rare complication. To date, 30 reports of such complications associated with pure stent knotting including our case have been reported since the first report in 1989.^3^, ^4^, ^5^ In the majority of cases, knotting was observed at the proximal end of the indwelling stent.

Urolithiasis is the main indication for ureteral stents. In other cases, retroperitoneal fibrosis, malignant ureteric obstruction, and ileal conduit have been reported as indications for stenting.^6^ In almost all reports, double‐J stent was inserted, and to the best of our knowledge, this is the first case report of the impaction of a knotted single‐J stent in patient with an ureterocutaneostomy.^3^

**Fig. 4:**
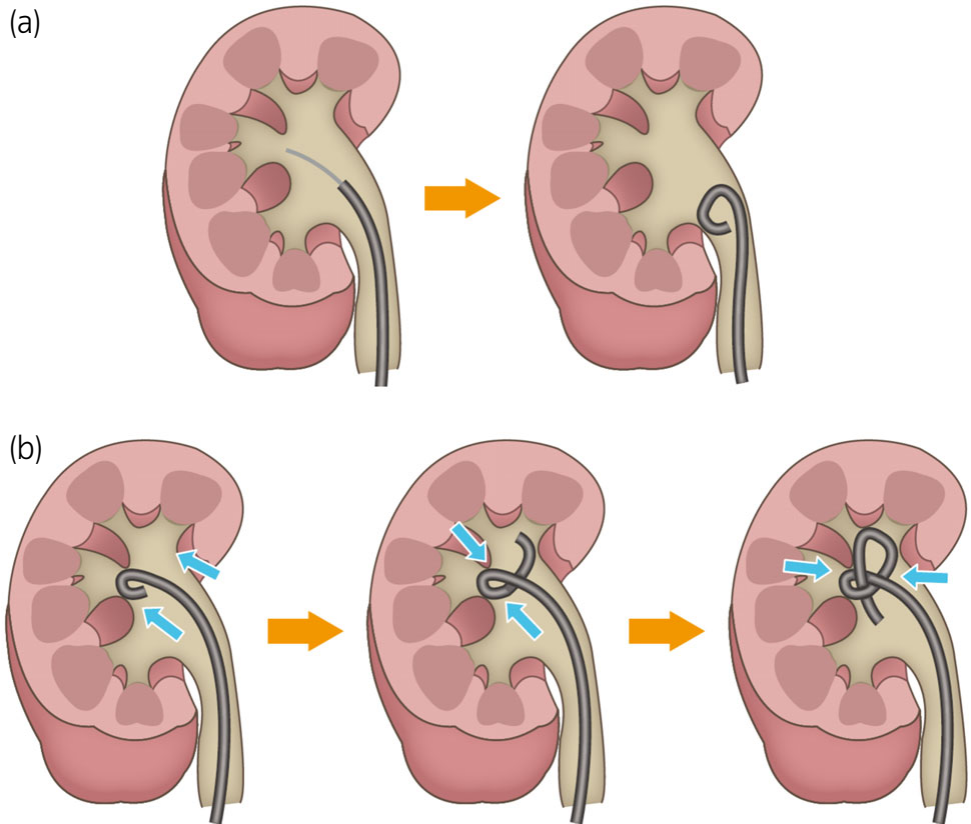
(a) Case with sufficient guide‐wire insertion during stent exchange. (b) Hypothesis of stent knot formation due to insufficient guide‐wire insertion during stent exchange.

Generally, knots occur during stent implantation or removal. However, the cause of knot formation in an indwelling ureteral stent remains controversial. An excessive length of the ureteral stent and anatomical factors of each patient have been hypothesized as causes of stent knotting.^3^ Although multi‐length stents are less likely to migrate in the ureter, they seem to have a higher risk of knotting; therefore, an optimal selection of stent length can prevent knotting.^7^ In our case, we used a single‐J stent whose coil length was shorter than the multi‐length and no trouble had been previously experienced with stent extraction. It is possible that the knot was formed because the guide‐wire was not fully inserted and the ring at the tip of the stent was pushed into the back while in contact with the renal pelvis (Fig. 4). Therefore, it is important to sufficiently insert the guide‐wire and straighten the stent. The specific situation in this case was that ureteral stent replacement was performed urgently by another urologist. The frequency of stent knotting has been reported to be influenced by the physician’s experiment.^8^ Although the exact cause of knot formation was not evident in this case, ureteral stent replacement should be performed with the help of imaging.

To date, the management of such a complication has no guidelines. Various techniques for the removal of knotted stents have been reported. Simple traction of the ureteral stent has been successfully demonstrated in approximately half of the cases.^3^ This procedure can be easily performed, but knot tightening can be achieved. Therefore, in case of resistance during extraction, other procedures should be considered. In other cases, successful removal of a knotted ureteral stent by grasping forceps or holmium laser fragmentation under ureteroscopy has been reported.^9^, ^10^ Although these procedures are minimally invasive, retrograde access to the knotted stent under ureteroscopy may be difficult in cases with ureteral stricture, and removal of fragmented stents can be difficult. Although nephroscopy is an invasive procedure, percutaneous removal of a knotted ureteral stent should be more reliable than the retrograde procedure.

Stent knotting is a rare complication that is difficult to treat in endourological practice, especially in case of solitary kidney.^11^ Percutaneous access is a reliable and safe alternative for the removal of such stents. In our case, as the patient had a solitary kidney, we need to avoid additional complications. In addition, as the patient is scheduled to undergo additional salvage chemotherapy, the stent should be managed without delay. Therefore, a percutaneous approach was decided.

In conclusion, we encountered a case of knotted ureteral single‐J stent. The removal of a ureteral stent should always be performed gently. In all cases in which resistance is experienced during extraction, the possibility of stent knotting should be considered and an appropriate removal strategy should be planned.

## Conflict of interest

The authors declare no conflict of interest.

## Approval of the research protocol by an Institutional Reviewer Board

Not applicable.

## Informed consent

Not applicable.

## Registry and the Registration No. of the study/trial

Not applicable.
